# Proton Pump Inhibitor Use, Hypergastrinemia, and Gastric Carcinoids—What Is the Relationship?

**DOI:** 10.3390/ijms21020662

**Published:** 2020-01-19

**Authors:** Denis M. McCarthy

**Affiliations:** Departments of Medicine & Biochemistry, Division of Gastroenterology and Hepatology, University of New Mexico School of Medicine and Raymond G. Murphy Veterans Administration Medical Center, 1501 San Pedro Blvd. SE, Albuquerque, NM 87108, USA; dmccarthy@salud.unm.edu

**Keywords:** proton pump inhibitors (PPI), gastrin, gastric carcinoid, neuroendocrine tumor (NET), gastric atrophy, Zollinger-Ellison syndrome (ZES), multiple endocrine neoplasia type-1 (MEN-1), *H. pylori*, ATP4A, MEN1, reg-1, CCK2R, netazepide

## Abstract

Neuroendocrine tumors (NETs) throughout the body are the focus of much current interest. Most occur in the gastrointestinal tract and have shown a major increase in incidence over the past 30 years, roughly paralleling the world-wide increase in the use of proton pump inhibitor (PPI) drugs. The greatest rise has occurred in gastric carcinoids (g-NETs) arising from enterochromaffin-like (ECL) cells. These tumors are long known to occur in auto-immune chronic atrophic gastritis (CAG) and Zollinger-Ellison syndrome (ZES), with or without multiple endocrine neoplasia type-1 (MEN-1), but the incidences of these conditions do not appear to have increased over the same time period. Common to these disease states is persistent hypergastrinemia, generally accepted as causing g-NETs in CAG and ZES, and postulated as having similar tumorigenic effects in PPI users. In efforts to study the increase in their occurrence, g-NETs have been classified in a number of discussed ways into different grades that differ in their incidence and apparent pathogenesis. Based on a large amount of experimental data, tumorigenesis is mediated by gastrin’s effects on the CCK2R-receptor on ECL-cells that in turn leads to hyperplasia, dysplasia, and finally neoplasia. However, in all three conditions, the extent of response of ECL-cells to gastrin is modified by a number of genetic influences and other underlying risk factors, and by the duration of exposure to the hormonal influence. Data relating to trophic effects of hypergastrinemia due to PPI use in humans are reviewed and, in an attached Appendix A, all 11 reports of g-NETs that occurred in long-term PPI users in the absence of CAG or ZES are summarized. Mention of additional suspected cases reported elsewhere are also listed. Furthermore, the risk in humans may be affected by the presence of underlying conditions or genetic factors, including their PPI-metabolizer phenotype, with slow metabolizers likely at increased risk. Other problems in estimating the true incidence of g-NETs are discussed, relating to non-reporting of small tumors and failure of the Surveillance, Epidemiology, and End Results Program (SEER) and other databases, to capture small tumors or those not accorded a T1 rating. Overall, it appears likely that the true incidence of g-NETs may be seriously underestimated: the possibility that hypergastrinemia also affects tumorigenesis in additional gastrointestinal sites or in tumors in other organ systems is briefly examined. Overall, the risk of developing a g-NET appears greatest in patients who are more than 10 years on drug and on higher doses: those affected by chronic *H. pylori* gastritis and/or consequent gastric atrophy may also be at increased risk. While the overall risk of g-NETs induced by PPI therapy is undoubtedly low, it is real: this necessitates caution in using PPI therapy for long periods of time, particularly when initiated in young subjects.

## 1. Introduction

Following a century of evolving terminology, gastric carcinoids, the term most used by clinicians and the subject of this paper, are now more accurately and definitively described as neuroendocrine tumors (NETs), a subgroup of gastro-entero-pancreatic endocrine tumors (GEPs), and hence formally designated GEP-NETs. For the purpose of this paper, however, I use the term “gastric carcinoid” (although archaic) to describe those NETS that are limited to the stomach, excluding those in the duodenum and small intestine, which may have a different pathogenesis [1,2]. Their proximate cells of origin appear to be enterochromaffin-like (ECL) cells, which like most NETs arise from locally multipotent gastrointestinal stem cells, and not from the neural crest as earlier hypothesized [3]. As NETs, they possess the capacity to synthesize and release various neuropeptides and amines, but unlike other NETs, their clinical importance lies more in their neoplastic potential than in any associated secretory syndrome.

**Figure 1:**
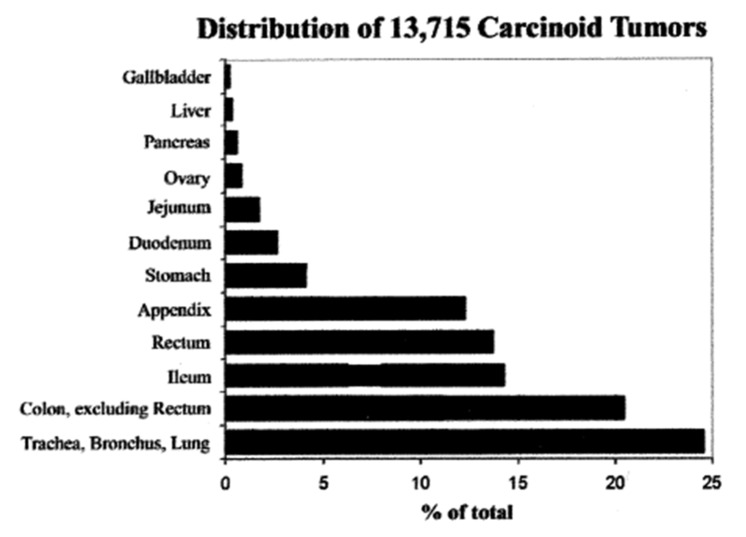
Sites of carcinoid tumors in the USA between 1950 and 1999 [6].

All NETs are the focus of much current attention. From the work of Dasari and colleagues, the following observations emerge [4]. Between 1973 (just before acid suppressive drugs became available) and 2012, a total of 64,971 NETs were recorded in North America, 52% of which occurred in women. Over this period, the age-adjusted incidence rates rose from 1.09 to 6.98 per 100,000-population, a 6.4-fold increase: this increase occurred across all sites, stages, and grades of tumor. The most dramatic rise was in patients 65 years and older, with a more than 8-fold rise to 25.3 per 100,000 population. Broken down by site, the greatest rise was in gastric tumors (15-fold), but rectal (9-fold), intestinal, broncho-pulmonary, pancreatic, and other carcinoids also showed marked increases in occurrence. The trend for an increase in the incidence of gastric carcinoids has also been noted in the UK over a similar time period [5]. The increase in gastric carcinoids is all the more impressive when examined against the background of the distribution of carcinoid tumors diagnosed over an earlier period of almost fifty years, most of which preceded the onset of the period of rapid increase [6]: over the earlier time, gastric tumors made up only 4.1% of all carcinoids (Figure 1). These striking changes suggest the recent emergence of an environmental factor that affected the incidence of all carcinoids, but particularly those in the stomach. Over this time period, the incidences of possibly confounding pathogenic influences, such as chronic atrophic gastritis (CAG)—with or without pernicious anemia, Zollinger-Ellison syndrome (ZES), or multiple endocrine neoplasia type-1 (MEN-1), all appear to have remained essentially unchanged.

In attempts to understand this change, a number of attempts at classifying gastric carcinoids into either 3 or 4 subtypes have been advanced [7,8]. There is also a World Health Organization (WHO) classification of NETs that groups them into well-differentiated Grade 1 and Grade 2 tumors (NET G1 and NET G2), and poorly differentiated Grade 3 tumors, neuroendocrine carcinomas (NEC G3), according to their mitotic or Ki-67 indices [9]. NETs with >30% atypical cells are arbitrarily regarded as carcinomas G3. In more recent classifications, tumors with a Ki-67 index <3% are designated as Grade 1, those with a Ki-67 index between 3% and 20% as Grade 2, and those with an index >20% as Grade 3 [10]. In these classifications, type-1 tumors are gastrin-dependent, sporadic, well-differentiated, arise in chronic hypochlorhydric states, comprise 70% to 80% of gastric carcinoids, and metastasize in <10% of cases [11]. Most are classified as Grade 1 (G1) on the WHO scale, with a Ki-67 index (if any) of <3%: they regress with resection of the gastric antrum, the location of the G-cells, which produce the gastrin. Type-2 gastric carcinoids occur in the presence of gastrinoma, primarily in those with MEN-1/ZES, rarely in uncomplicated sporadic ZES, comprise 5%–6% of gastric carcinoids, are gastrin-dependent, and malignant in 10%–30% of cases [11]. Type-3 tumors are gastrin-independent, sporadic, well-differentiated, comprise 14%–25% of gastric carcinoids, and 25%–40% are malignant. Type-4 tumors are sporadic, gastrin-independent, poorly differentiated, and metastasize in 50%–100% of cases [11]. There are rare gastric carcinoids that do not fit into this classification [12]. It is believed that virtually all carcinoids arise from ECL cells that possess gastrin/CCK2R receptors; in types-1 and 2 these cells are stimulated to proliferate by chronic hypergastrinemia, sequentially undergoing hyperplasia, dysplasia, and carcinoid formation, with or without progression to malignancy [13]. Validating the role of gastrin, type-1 lesions show complete regression during treatment with the gastrin/CCK2R-receptor antagonist netazepide [14,15], but the regression does not persist when treatment ceases, so type-1 NETs recur gradually because the presence and action of hypergastrinemia is unchanged by therapy with netazepide.

In the case of type-2 tumors, in patients with MEN-1, a hereditary condition caused by mutations which inactivate the tumor suppressor gene *MEN1*, the tumorigenic influence of gastrin may be accentuated by reduced tumor suppression, an additional factor in inducing carcinoids [16], although it must also be pointed out that despite the fact that using villin-Cre to delete the *MEN1* locus in the gastrointestinal epithelium generated hypergastrinemia, G-cell hyperplasia and epithelial dysplasia, no ECL tumors developed [17]. This suggests that more than one alteration to this genome may be required for the genesis of type-2 NETs in MEN-1, or that deletions or heterozygosity in the somatostatin genome may also be involved [18,19]. However, in MEN-1 patients, type-2 carcinoids also regress after excision of all gastrinomas and serum gastrin has returned to normal [20]. The extent to which downstream proliferative cellular responses to ECL-cell secretion of Reg-1 protein is responsible for tumorigenic/carcinogenic effects on gastric mucosa remains uncertain [21].

The effects of hypoacidity and consequent hypergastrinemia on gastric neoplasia have been reviewed in detail elsewhere, including results from a wide variety of experiments in animal models [11,13,22,23]. Of particular note, the prolonged use of proton pump inhibitors (PPIs) or of an insurmountable H2–antagonist loxtidine, induced malignant ECL-derived tumors in the oxyntic mucosa of rodents. The results in all these studies supported the hypothesis that prolonged hypochlorhydria caused hypergastrinemia, which in turn caused ECL-cell proliferation, dysplasia and neoplasia: proliferations regressed when hypergastrinemia ceased. 

Human clinical conditions causing hypergastrinemia include the hyperchlorhydric state caused by gastrinoma in Zollinger-Ellison syndrome (ZES), with or without MEN-1, and the hypochlorhydric states of chronic atrophic gastritis (CAG) due to autoimmune gastritis/pernicious anemia or *Helicobacter pylori* infection, vagotomy with gastric resection (some retained antrum), and prolonged proton pump inhibitor therapy. In a rare human disease, that closely resembles exposure to prolonged PPI therapy, members of a Spanish family, homozygous for an inactivating mutation in the gene ATP4A that regulates expression of the alpha subunit of H^+^/K^+^ ATPase, have the inability to secrete gastric acid and consequently have life-long hypochlorhydria and hypergastrinemia [24,25]. Affected members may develop both gastric NETs and gastric neuroendocrine carcinomas (NECs) that show immune-reactivity for the neuroendocrine markers chromogranin A (CgA) and synaptophysin, as well as for the ECL-specific markers, vesicular monoamine transporter (VMAT2), and histidine decarboxylase (HDC) [24]. However, in affected cases, tumors did not appear until the fourth decade of life, indicating that hypergastrinemia had to exist for a long time before tumors developed. A very rare case reported in Japan, in the era prior to genomic characterization but after the introduction of PPIs, may have a similar etiology, a possibility raised by the authors [26]. This background brings us to examine the essential question, whether or not prolonged hypergastrinemia due to PPI therapy induces gastric carcinoid formation in humans? 

## 2. Observations in Humans

Long-term human use of PPIs leads to hypergastrinemia in almost all cases, with fasting serum gastrin (FSG) levels increasing 3 to 5-fold in most subjects, but to much higher levels in 10% to 30% of cases [11]. Higher levels are seen in females and in those with atrophy of the gastric corpus due to CAG, whether auto-immune or due to *Helicobacter pylori* [27]. Recent studies, however, have shown that FSG levels in PPI users do not reflect the 4- or 5-fold elevations in mean serum gastrin concentrations that occur and persist after meals, particularly in females [28]. These results indicate that studies to date may have seriously underestimated ECL-cell exposure to gastrin in PPI users. Furthermore, previous normal gastrin limits are too high since many of the persons defined as normal had *Helicobacter pylori* gastritis, when the normal values were determined, before recognition of the existence of *Helicobacter pylori* [28].

Hypergastrinemia leads to proliferation and hyperfunction of ECL-cells in man and animals [22,23]. There is no lower threshold for the ECL-specific, trophic effect of gastrin, but it is concentration dependent, and closely associated with long term exposure or use [29,30,31]. The effect is mediated by gastrin/CCKB2R receptors on the ECL-cell membrane, and the maximum trophic effect on the ECL-cell is achieved by a plasma concentration of 500 pmol/L, above which a plateau is reached [30,32]. Stimulation by hypergastrinemia causes the ECL-cell to proliferate and secrete chromogranin A, a commonly used biomarker for the presence of NETs, including gastric carcinoids, but also elevated by the hypergastrinemia that accompanies PPI use: it is thus of limited value due to poor specificity [33,34]. Chromogranin A is specific for neuroendocrine cells but does not discriminate between hyperplasia and neoplasia. It is good as a substitute marker for 24-h gastrin exposure. In the context of gastric carcinoids, a marked fall, when the tumor has been excised and PPI discontinued, is good evidence of complete removal, while a failure to fall may indicate metastatic spread.

## 3. Evidence of Causation

There are now eleven cases reported in whom gastric carcinoid (g-NET) tumors developed while the patient was using a PPI, and in whom evidence of ZES/MEN-1 or gastric oxyntic mucosal atrophy was lacking [12,35,36,37,38,39,40,41]. In the interests of space, brief summaries of each case are attached as an Appendix A. The serious reader is advised to consult the original reports. These summaries are presented because many of the details they contain have been glossed over by reviewers who were primarily concerned with the overall clinical safety of PPIs. However, what emerges, from reading these case reports in detail, is a convincing pattern of evidence that hypergastrinemia caused by PPIs stimulated ECL-cell hyperplasia, similar to that seen in animals, and at least in a subpopulation of humans, the hyperplasia progressed to tumors of various kinds, especially type-1 gastric carcinoids (although the original classification did not include PPI use as a cause of type-1 lesions).

From the earliest report [35] in 1998, there has been progressive expansion in the amount of detail provided on each case that allows increasingly critical evaluation of the evidence presented. Review of each case by this author, in the light of current knowledge, may not agree with the conclusion of the original author(s) in all cases: for instance, while Appendix A case #3 [37] was alleged to occur in a patient with pernicious anemia (an obvious confounder), other details presented in the publication cast serious doubt that this condition was present. Similarly, while authors of case #7 [40] discussed classifying that case as a type-3 g-NET, this was invalid because serum gastrin was not measured before excising the tumor and discontinuing PPI therapy: from other described cases serum gastrin would be expected to be normal by the time the patient was tested, thus defying accurate classification.

Despite minor differences in classifying these 2 cases, there are 11 cases who appear to have developed g-NET tumors following mostly long-term therapy with PPIs. Only 1 was malignant and most of them were well-differentiated. Given that the animals developing NETs and NECs have often been exposed to drug for most of their life span, and that humans in whom ATPase is congenitally absent, and who experience life-long hypochlorhydria, do not develop such tumors until the third or fourth decade of life [22,23,24,25], it is not surprising that long exposure to the drug may be a major requirement for tumor development. This is an important clinical consideration in young patients with severe gastro-esophageal reflux disease who may need to use potent acid suppressants for prolonged periods: conversely, the carcinoid risk may be negligible in patients beginning such therapy in later life. Other factors may emerge that specifically identify subpopulations at an enhanced risk of developing PPI-associated carcinoids including: presence of chronic gastritis (short of corpus atrophy) due to autoimmunity or *H. pylori* infection—the latter perhaps accelerated by PPI therapy [22,42,43]; the dose or duration of PPI therapy; the presence of a PPI slow-metabolizer phenotype; or the presence of mutations in other genes e.g., MEN-1 or those affecting somatostatin responses. However, given the low incidence of carcinoid occurrence in all PPI users, identifying such factors with certainty may take a long time. The need for long exposure to the drug also explains the failure of this risk to emerge in studies of up to 10-years of duration of PPI therapy, even in very large population samples [11,31,44]. Finally, there are some differences between the reported cases in the magnitude of: fasting serum gastrins; serum chromogranin A levels; extent of ECL-cell hyperplasia; and type of carcinoid, although most are type-1 G1 (see Appendix A). 

Related to risk, the exact incidence rate of PPI-induced NETs is somewhat uncertain and may be underestimated. Apart from the 11 cases reported here, there are additional, not fully described cases in a previously mentioned United States Food and Drug Administration report, a minority of which (based on dates of occurrence) may also be reported here [45]. One carcinoid was reported in long-term omeprazole trial 017 [45]. In long-term trials prior to June 1998, there were 6 gastric carcinoids not attributable to gastrinoma or pernicious anemia [45]. Among post-marketing surveillance reports prior to 2000, there were 13 carcinoids not attributable to gastrinoma or other conditions, 9 of which were gastric and 4 duodenal [45]. Incidence data based on data from the SEER registry may also be underestimated, because until more recently, incident cancers in many states were reported to SEER only if the tumor was malignant [4]. That this could be an important source of error emerges from a study by Hodgson et al. from Florida, in which all carcinoids were reported, benign or malignant, over a 20-year period [46] with 26 gastric carcinoids in the state. Over the same time, the SEER database recorded a total of 594 from 9 states, an average of 66 cases per state, roughly 20% of the incidence rate in Florida. It is very doubtful that Florida has an incidence rate of gastric carcinoids 5 times that of 9 other states. The discrepancy, however, suggests that there was serious under-reporting of gastric carcinoids in many of the states reporting to SEER. Thus, the estimated US incidence rate of gastric carcinoid of 0.42 per 100,000 (2012) could be as much as 5-fold higher [4]. While reporting is likely more accurate in recent years, the exact incidence is unknown but likely higher: many pathologists do not attach a T1 tumor designation to NETs <5 mm in size, so many will escape being recorded. This may explain why carcinoids were found in approximately 1% of necropsies, even before the era of PPI use [47]. Despite these limitations, available data are sufficient to demonstrate that a major increase in the reported incidence of gastric carcinoids has occurred over the same time period as the increased use of PPIs [42,48]. The cases reported here, occurring in the absence of gastrinoma or gastric atrophy, are sufficient to warrant acceptance of these time trends being causally connected i.e., long-term use of PPIs is a major factor contributing to the rise in gastric carcinoids, many of which regress when PPI therapy is discontinued. 

**Figure 2:**
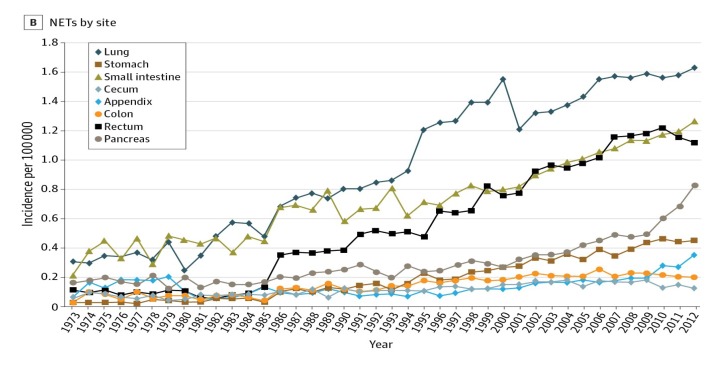
Incidence trends of neuroendocrine tumors (NETs) from 1973 to 2012 [4].

While gastric carcinoids, despite their marked increase, occur much less frequently than carcinoids in the lung, rectum, small intestine, and pancreas, these other tumors also display marked increases in their occurrence over the same time period (Figure 2), although serum gastrin has not been studied in affected patients [4,11]. 

Furthermore, studies suggest that not only stem cells but also mature neuroendocrine cells, upon long-term over stimulation by gastrin, may progress through hyperplasia and dysplasia into highly malignant carcinomas [49]. Together, these observations suggest that by causing hypergastrinemia, widespread use of PPIs, often for many years and particularly in patients with intractable reflux disease, may be contributing to increase in the occurrence of many cancers, both within and outside the gastrointestinal tract. The true extent of possible extra-intestinal stimulation has not been studied, but examination of elevations of serum chromogranin A has been associated with the presence of tumors of pituitary, parathyroid, thyroid, medullary thyroid, adrenal, lung small cell, prostate, and neural (Schwannoma) origin [34], suggesting that growth in these also may possibly be stimulated by hypergastrinemia.

Of particular relevance is the possible effect of PPI use on gastric cancers. While a detailed review of the role of PPIs in influencing the development of gastric adenocarcinomas, both those in the region of the cardia and infiltrative tumors regardless of location, is beyond the scope of this article, it is, however, the subject of great current interest and extensively reviewed elsewhere [23,50,51]. Regardless of mounting evidence from laboratory experiments, following the systematic review of Tran-Duy in 2016 [52], who analyzed pooled data from 12 studies comprising 87,324 patients, and showed an odds ratio of 1.46 (95% confidence interval 1.23 to 1.66) for association, there is increasing attention being paid to examining the effect of PPIs on gastric cancer in men. A more recent major study [53] that included virtually all adults in Sweden, exposed to maintenance therapy with PPIs for more than 180 days (total 797,067 subjects), showed a standardized incidence ratio (SIR) of 3.38 (95% confidence interval, 3.23 to 3.53) and included all ages and both sexes. The association was especially increased among those under age 40 years (SIR 22.76, 95% confidence interval 15.94 to 31.52): the use of H_2_-receptor antagonists was not associated with an increased risk [53]. The reason for this increased risk remains the subject of discussion, but there seems little doubt of the existence of an important linkage between PPI use and gastric cancer. Finally, it may be that there is a significant effect of hypergastrinemia not only on various types of neoplasia in the stomach, but possibly also on growth of tumors (especially endocrine) in others sites.

## 4. Summary

Gastric carcinoids are members of a family of principally gastro-entero-pancreatic (GEP) neuroendocrine tumors (NETs), although related similar tumors also occur in other organs. The incidence of gastric carcinoids has increased up to 15-fold over the past 3 decades, a period characterized by a parallel major increase in the use of acid-secretory-suppressing proton pump inhibitor drugs. This increase occurred in a type of carcinoid, which prior to this period, comprised only 4% of all reported carcinoids. Over the same period, there was no discernible increase in other known and potentially confounding causes of this tumor. The increase in incidence has gradually led to development of various tumor classifications. These allow an increasingly precise definition of various types of NETs, identification of various pathogenetic origins, and study of the circumstances under which they arose. While the trophic effect of gastrin leading to ECL cell hyperplasia may be a necessary factor in increasing tumor development, it may not be the sole factor involved; loss or impaired expression of the tumor-suppressor gene menin (even in heterozygous subjects), dysregulation of somatostatin response genes or those regulating chromogranin A, Reg-1 protein expression, phenotype for PPI-degradation, or presence of acquired diseases such as *H. pylori* or auto-immune chronic gastritis, may all modify the effects of PPIs in different susceptible individuals.

Extensive laboratory research in animals has provided evidence that hypergastrinemia, regardless of cause, leads to oxyntic ECL-cell hyperplasia that in a minority of cases progresses through dysplasia to neoplasia. That this applies also in humans is directly supported by the occurrence of gastric NETs and NECs in humans experiencing life-long hypochlorhydria, due to their lacking the ATP4A gene that codes for H^+^/K^+^ ATPase, the enzyme essential to gastric acid secretion in man. This state of life-long hypochlorhydria is the state most closely resembling the effect of prolonged exposure to PPI therapy, and is supported by the development of gastric carcinoids in a number of patients treated with PPIs over many years: relevant data on these is appended.

That the 11 cases and the tumors are not all identical is probably reflective of different modifying influences, both genetic and environmental, the latter including *H. pylori* or autoimmune gastritis; host pharmacokinetics; type, dose, and duration of PPI therapy: and other as yet unrecognized factors. That NETs as a complication of PPI therapy, are slow to develop, and rare in occurrence, is beyond dispute, but for reasons reviewed, the true incidence of their development is probably underestimated. The PPI-induced, growth-promoting effects of gastrin, on tumors in the stomach, increasingly appears to affect gastric cancers as well as gastric carcinoids, and may well extend to cancers in other organs. That there is widespread, excessive, chronic use of PPIs world-wide is generally accepted, and the overall impact of this on tumorigenesis remains unknown, but the effect of long-term PPI therapy, on stimulating the rare development of at least type-1 gastric carcinoids, seems beyond dispute. This is not to imply that, as a class of clinically very useful drugs, PPIs are not safe and effective when used judiciously for a moderate time.

## Appendix A. Gastric Carcinoids Associated with the Use of PPI: Summary of Features of Reported Cases

# 1. A 31 yr-old male, over a 6-yr period had 4 courses of therapy for duodenal ulcer (35 months exposure to acid suppression). He developed a single type-1 carcinoid in the body of a non-atrophic stomach with prominent folds. Concentrations of serum gastrin, chromogranin, *Helicobacter pylori* status, or gastric autoantibodies were not measured, and gastric mucosa was not biopsied. He did not appear to have ZES or MEN-1. He was treated with surgical excision and selective proximal vagotomy, with no recurrence of ulcer or carcinoid during 32 months of follow-up [35].

# 2. A 56 yr-old female taking PPI for 4 years, who developed a single type-1 carcinoid in the body of the stomach, had a fasting serum gastrin of 128 pmol/L (normal 0–40): fell to 40 pmol/L after anti-reflux surgery and stopping PPI therapy: no other was information provided [36].

# 3. A 52 yr-old female, on PPI “for several years”, developed multifocal type-1 gastric carcinoids, with a serum gastrin of 1111 pg/mL (range 0–100 pg/mL) and serum chromogranin of 54 ng/mL (normal 6–39 ng/mL). Based on the finding of anti-gastric parietal cell antibodies (titre1: 320) she was labelled as having pernicious anemia, but anti-intrinsic factor anti-bodies were negative, intra-gastric pH was 2.0, and gastritis was antral, suggestive of *H. pylori* infection, although gastric biopsies were negative for the organism. Histochemichal staining of tumor biopsies revealed the presence of chromogranin A and synaptophysin. No follow–up data was provided [37].

# 4. A 49 yr-old male, using PPI for 15 years, developed a gastric carcinoid in a hiatal hernia sack, containing normal non-atrophic oxyntic mucosa adjacent to the tumor. Serum gastrin was 149 nmol/L (normal < 40 pnmol/L) and chromogranin 32 nmol/L (normal < 6 nmol/L) with mucosal biopsies negative for *Helicobacter pylori*. On immunolabeling with H^+^/K^+^ ATPase, biopsies of the oxyntic “mucosa showed abundant parietal cells; staining with monoclonal antibody against CgA disclosed linear enterochromaffin-like (ECL) hyperplasia, indicating long-term hypergastrinemia. Additional staining of the excised tumor revealed the presence of synaptophysin, CgA, vesicular monoamine transporter 2 (VMAT 2), and positivity on Sevier-Mungar staining, all indicating an ECL cell origin. However, the Ki-67 index was 90%, the histology was that of a poorly differentiated tumor, and an involved lymph node was also found. The best classification would seem to be that of a malignant type-1 carcinoid [38].

# 5. A 55 yr-old male, using PPI for 12 years was found at upper gastrointestinal endoscopy (UGE) to have an 18 mm tumor in the gastric corpus. Gastrin or chromogranin were not measured while on PPI but, 20 months post resection and stopping PPI, were normal, serum gastrin 5 pmol/L and chromogranin 4 nmol/L. Tests for *H. pylori* were negative. Median intragastric pH was <4.0 for 82% of the time, thus excluding atrophic gastritis. The tumor, on staining, revealed the presence of chromogranin A and VMAT 2, and Ki-67 was <2%: it is classified as a type-1 gastric carcinoid [39].

# 6. A 68 yr-old female with a 15 mm tumor in the gastric corpus at UGE, had been on PPI for 13 years. Serum gastrin (191 pmol/L) and serum chromogranin (19.7 nmol/L) were both elevated while taking PPI, but both normalized 3 months after drug use ceased. She had *H. pylori* found in biopsies and this was treated to eradication. Intragastric pH was <4.0 for >84% of the time eliminating the presence of gastric atrophy. The tumor was biopsied but not fully excised. PPI therapy was stopped and the tumor disappeared 2 to 3 months later [39]. Microscopic examination of the biopsies showed a well differentiated NET, positive for chromogranin A and VMAT-2, and with Ki-67 <2%. Gastric mucosa was hypertrophic with abundant parietal cells and pronounced linear and micronodular ECL-cell hyperplasia, the usual results of chronic hypergastrinemia. Three months after stopping PPI, both serum gastrin and chromogranin A had returned to normal and ECL-cell hyperplasia had disappeared, but diffuse ECL-density was still present. This is essentially a benign type-1 gastric carcinoid [39].

# 7. A 48 yr-old female, using PPI for 8 years for esophageal reflux disease, at UGE had 3 unremarkable fundic polyps, in addition to an unusual 10 mm pedunculated polyp in the region of the gastric cardia. Serum gastrin or serum chromogranin do not appear to have been measured before either removal of this polyp or cessation of PPI therapy, but both levels were normal 1 month after this. The polyp was removed. Later, following revision of the pathology report, it was diagnosed as a gastric carcinoid. At a subsequent UGE, the fundic polyp were removed and proved to be typical cystic glandular fundic polyps commonly seen in PPI users. Histological examination of multiple gastric biopsies diagnosed corpus gastritis without signs of active inflammation, atrophy, pyloric, or intestinal metaplasia. The patient was negative for *H. pylori* infection. The Ki-67 was 1%. Despite some confusing aspects of the investigation and discussion, and the lack or omission of several relevant test results, the polyp appears to have been a benign type-1 carcinoid [40].

# 8. A 76 yr-old male using PPI for 15 years for relief of esophageal reflux disease was referred for investigation after the finding of a 4 mm g-NET, a polypoid mass, covered by normal mucosa which was endoscopically resected. Histological examination revealed a well-differentiated NET (Ki-67 <1%. Multiple gastric biopsies showed chronic antral gastritis, without atrophy or *H. pylori* infection. Fundic biopsies showed ECLO-cell hyperplasia. Serum gastrin was markedly elevated (1250 pg/mL: normal <108) and serum chromogranin A was also mildly elevated (29 U/L; normal < 20) anti-parietal cell, *H. pylori* IgG and IgM antibodies were all negative. Cessation of PPI therapy resulted in normalization of serum gastrin, and at UGE 12 months off drug, there was complete reversal of ECL-cell hyperplasia. At 12 month and 24 month endoscopic follow up, there was no sign of g-NET recurrence or of chronic atrophic gastritis. This was a type-1 gastric carcinoid [41].

# 9. A 58 yr-old male using PPIs for at least 5 years for esophageal reflux, underwent UGE for worsening symptoms, which lead to discovery of a 5 mm polyp in the gastric body. Histology revealed a well differentiated NET but with a Ki-67 of 4% and a mitotic count of 3/10 HPF (NET type-1, G2). There was some evidence of lympho-invasion but no angio-invasion. There was ECL-cell hyperplasia throughout the antrum, body, and corpus, and mild inflammation in the corpus but without atrophy. Cystic gland polyps were observed in the gastric body and fundus. Staining for *H. pylori* was negative. Fasting serum gastrin concentration was within normal limits but serum chromogranin A level was elevated (71 U/L; *n* < 20 U/L). Gastric parietal cell, *H. pylori* IgG and IgA antibodies were all normal. Serum chromogranin and gastrin levels fell to normal once PPI was discontinued. Twelve months after endoscopic removal of the tumor, there was no recurrence of the g-NET nor signs of ECL-cell hyperplasia or chronic atrophic gastritis. Bearing in mind the low risk of malignant change, associated with such a low Ki-67, this was still, by definition, a type-1 G2 NET [41].

# 10. A 66 yr-old female using PPIs for >15 years had UGE for worsening symptoms of esophageal reflux, and was found to have an elevated, flat, 15 mm g-NET in the gastric fundus. The lesion, being atypical, was extensively biopsied and revealed well differentiated G-Net G1 with a Ki-67 < 2%. Biopsies from the antrum body and fundus showed mild chronic inflammation, devoid of atrophy or *H. pylori* infection, but with some cystic polyps in the fundus, as often seen in PPI users, but there was no ECL-cell hyperplasia. There were mild elevations in serum gastrin (136 pg/mL: normal < 108) and chromogranin (47 U/L: *n* < 20) which returned to normal 1 month after PPI was discontinued. Screening for anti-gastric parietal, *H. pylori* IgG and *H. pylori* IgM antibodies were all negative. Endoscopic ultrasound demonstrated a submucosal lesion 1.8 cm in diameter and the patient elected to have a total gastrectomy. Histology of the specimen, but one pathological lymph node, was positive out of 15 examined. PPI was discontinued and follow-up surveillance with biochemical, endoscopic, and radiologic imaging is planned. At 6 months the gallium-PET scan showed no g-NET recurrence. This tumor was atypical but given hypergastrinemia was more akin to a type-1 G2 [41].

# 11. A 78 yr-old male with esophageal reflux disease, using PPI for over 20 years, underwent an UGE, which revealed a 3 cm ulcerated nodular mass in the gastric corpus, which on biopsy, revealed a tumor infiltrating the oxyntic mucosa with an organoid growth pattern, forming nodules 2 mm in diameter that stained strongly for chromogranin. Stains of these biopsies were all negative for gastrin. Positivity for chromogranin but not gastrin in a biopsy from oxyntic mucosa strongly suggests that these were ECL-cells but staining for synaptophysin or specific ECL marker e.g., histidine de-carboxylase or VAMT 2 were not available or performed. The appearances were those of a well-differentiated g-NET. Stains of adjacent gastric mucosa revealed no active gastritis or *H. pylori* organisms, serum anti-gastric parietal cell or intrinsic factor antibodies were negative, and serum B12 was normal. Fasting serum gastrin was 752 pg/mL (*n* < 115 pg/mL). A subsequent EGD with extensive gastric mapping revealed normal oxyntic mucosa, diffuse parietal cell hyperplasia and hypertrophy, but surprisingly no ECL-cell hyperplasia (no chromogranin staining). The tumor was removed by laparoscopic wedge resection and was strongly positive for chromogranin. It originated in the submucosa but did not meet criteria for ECL-cell malignancy. Five months after resection and stopping PPI, serum gastrin had fallen to 37 pg/mL (*n* < 108 pg/mL). However, a primary care physician, in error, then restarted PPI therapy and 6 months later (11 months post resection) serum gastrin had again risen to 357 pg/mL. Classification of this tumor is problematic. It somewhat resembles a type-3 NET being large and solitary but found in the apparent absence of ECL-cell hyperplasia of the oxyntic mucosa. On the other hand, it was well differentiated, occurred in the presence of PPI-induced hypergastrinemia and profound parietal cell hyperplasia, and in the absence of MEN-1, ZES or gastric atrophy due to auto-immune or *H. pylori*-induced chronic gastritis. These features are suggestive of a type-1 GI g-NET. The apparent origin in the submucosa is unique [11].
